# Three new species of *Illiesonemoura* Baumann, 1975 (Plecoptera, Nemouridae) from China

**DOI:** 10.3897/BDJ.11.e112020

**Published:** 2023-11-14

**Authors:** Zhi-Jie Wang, Yu-Zhou Du, Xiao-Yu Ji

**Affiliations:** 1 Suqian University, Suqian, China Suqian University Suqian China; 2 School of Horticulture and Plant Protection & Institute of Applied Entomology, Yangzhou University, Yangzhou, China School of Horticulture and Plant Protection & Institute of Applied Entomology, Yangzhou University Yangzhou China

**Keywords:** aquatic insects, taxonomy, morphology, *I.bituberculata*, *I.motuoensis*, *I.weii*

## Abstract

**Background:**

The genus *Illiesonemoura* Baumann, 1975 (Plecoptera, Nemouridae) is a small-sized stonefly with slender and curved embranous cerci. Currently, 18 species of the genus are known worldwide, mainly distributed in the Palaearctic and Oriental Regions, with a total of two species known to China.

**New information:**

Three new species of *Illiesonemoura* Baumann, 1975, *I.bituberculata* Wang & Du, sp. nov., *I.motuoensis* Du & Ji, sp. nov. and *I.weii* Du & Ji, sp. nov. are described and illustrated, based on male adults from China. *Illiesonemourabituberculata* is characterised by two pairs of tubercles arising posteromedially from tergum 10 and by two rows of spinules outlining the lateral edge of the ventral sclerite of the epiproct. *Illiesonemouramotuoensis* is characterised by the heart-shaped epiproct with a thin and slightly sclerotised protrusion between the sclerotised bands. *Illiesonemouraweii* is characterised by a pair of small knobs on tergum 10, outer lobes of paraprocts basally broad, then slender towards apices with a pointed tip and the epiproct with lateral spinules.

## Introduction

The family Nemouridae is one of the common small stoneflies in the Northern Hemisphere (Baumann 1975) and it is the most species-rich family in the order Plecoptera (RE et al. 2023). The genus *Illiesonemoura* is a relatively small group in Nemouridae, comprising only 18 species known worldwide. The majority of these species are predominantly recorded in East Asia, South Asia and Central Asia (Baumann 1975, Zhao et al. 2023, RE et al. 2023). *Illiesonemoura* shares a similar living environment with other nemourid genera. They rather prefer running clear and cool streams or lakes. They are often collected from mountain streams, springs, creeks, from low flatlands, small to large, to high mountain streams and almost stagnant to rapid waters. Nymphs are primary consumers and feed mostly on detritus (Baumann 1975).

Baumann (Baumann 1975) proposed the genus *Illiesonemoura* and designated *Nemourapunctata* Jewett, 1958 as its type species. Another 17 species from *Nemoura* were transferred into *Illiesonemoura* later (Wu 1962, Aubert 1959, Harper 1975, Jewett 1958, Kawai 1968, McLachlan 1875). Two of these species, *I.tuberostyla* (Wu 1962) and *I.bispinosa* (Kawai 1968) occur in China. However, it is important to note that *I.bispinosa* was recently re-assigned back to *Nemoura* genus by Mo et al. (Mo et al. 2020). Furthermore, a new species of *Illiesonemoura*, *I.qianae* Du, Zhao & Rehman 2023, was recently documented in China (Zhao et al. 2023). As a result, there are currently two recognised species of this genus in China.

Tibet is an autonomous region in southwest of China, bordered by Sichuan to the east, Yunnan to the southeast and Nepal and other countries to the south and west. The Himalayan high mountain area is located in southern Tibet. Sichuan is a province in southwest China, close to the east of Tibet. However, there are still many areas in western China that lack further research on the diversity of the order Plecoptera. The lower sampling rate may lead to underestimation of species diversity in *Illiesonemoura*. In this paper, the specimens of stoneflies from Sichuan Province and Tibet, which had not been reported before, were sorted out and studied and three new species of the genus *Illiesonemoura* are described and illustrated (Fig. 12).

## Materials and methods

The specimens were collected by sweep-net from plants on both sides of a clean water stream (width 10 m) in Sichuan Province and Tibet. All specimens used in this study were preserved in 75% ethanol. Photos were taken with a KEYENCE VHX-5000 system and optimised by Adobe Photoshop CS6. The illustrations were made by hand and Adobe Photoshop CS6. The specimens are deposited in the Insect Collection of Yangzhou University (ICYZU), Jiangsu Province, China. Only male specimens were caught, so females remain unknown. Terminology follows that of Baumann (1975).

## Taxon treatments

### Illiesonemoura bituberculata

Wang & Du sp. nov.

1D8A9AA9-8566-50E3-AAFF-20D23B81EBB3

4C37F05B-C7FB-4DF7-A31D-CDE5D04C1CF2

#### Materials

- **Type status:** Holotype. **Occurrence:** recordedBy: Yu-zhou Du; individualID: Insect collection of Yangzhou University (ICYZU), Jiangsu Province, China; individualCount: 1; sex: male; lifeStage: adult; occurrenceStatus: present; occurrenceID: 82D320BE-8137-54E6-B600-64B17A0A86DE; **Taxon:** kingdom: Animalia; phylum: Arthropoda; class: Insecta; order: Plecoptera; family: Nemouridae; genus: Illiesonemoura; specificEpithet: *bituberculata*; **Location:** continent: Asia; country: China; countryCode: CN; stateProvince: Sichuan; locality: 15 km west of Kangding, Zheduo River; minimumElevationInMeters: 2920; verbatimLatitude: 29.994447N; verbatimLongitude: 101.892415; **Identification:** identifiedBy: Zhi-jie Wang, Yu-zhou Du & Xiao-yu Ji; **Event:** year: 1996; month: 6; day: 8; **Record Level:** language: en
- **Type status:** Paratype. **Occurrence:** recordedBy: Yu-zhou Du; individualID: Insect collection of Yangzhou University (ICYZU), Jiangsu Province, China; individualCount: 1; sex: male; lifeStage: adult; occurrenceStatus: present; occurrenceID: 7053928D-FDFD-51B3-AF16-C74A29F497D6; **Taxon:** kingdom: Animalia; phylum: Arthropoda; class: Insecta; order: Plecoptera; family: Nemouridae; genus: Illiesonemoura; specificEpithet: *bituberculata*; **Location:** continent: Asia; country: China; countryCode: CN; stateProvince: Sichuan; locality: 15 km west of Kangding, Zheduo River; minimumElevationInMeters: 2920; verbatimLatitude: 29.994447N; verbatimLongitude: 101.892415; **Identification:** identifiedBy: Zhi-jie Wang, Yu-zhou Du & Xiao-yu Ji; **Event:** year: 1996; month: 6; day: 8; **Record Level:** language: en

#### Description

**Figure 1.**
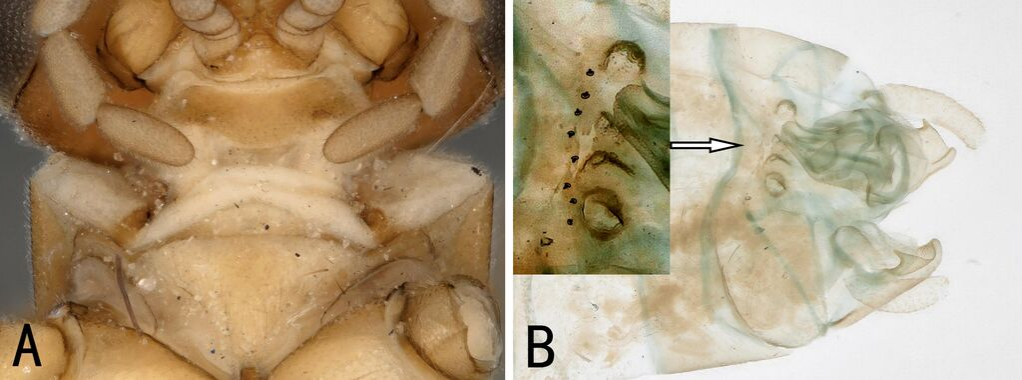
Male of *Illiesonemourabituberculata* sp. nov. **A** cervical region in ventral view; **B** terminalia in lateral view.

**Figure 2.**
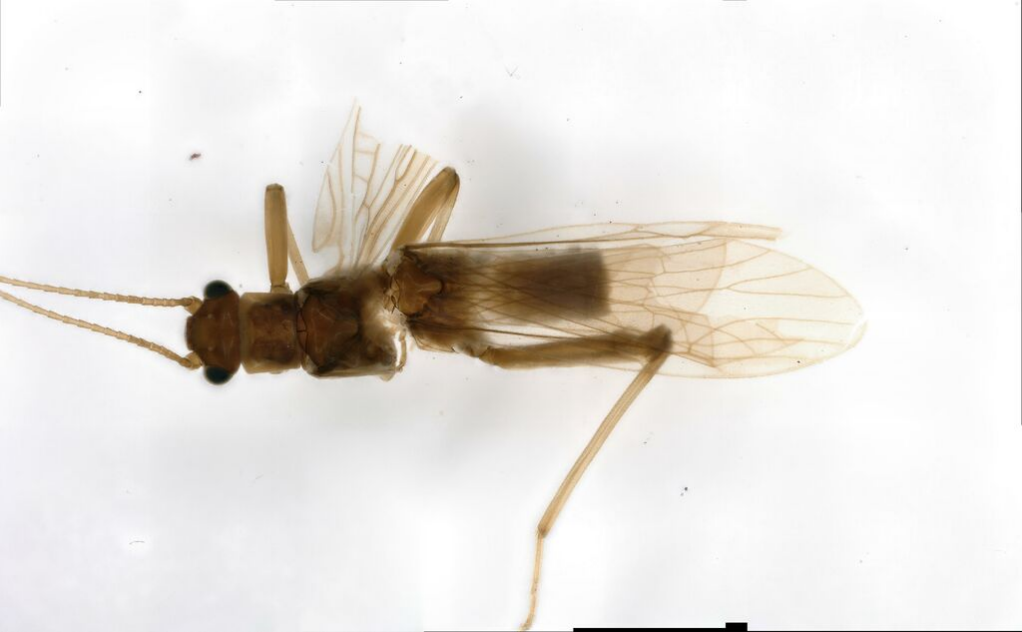
Male of *Illiesonemourabituberculata* sp. nov. Adult, dorsal view.

**Figure 3.**
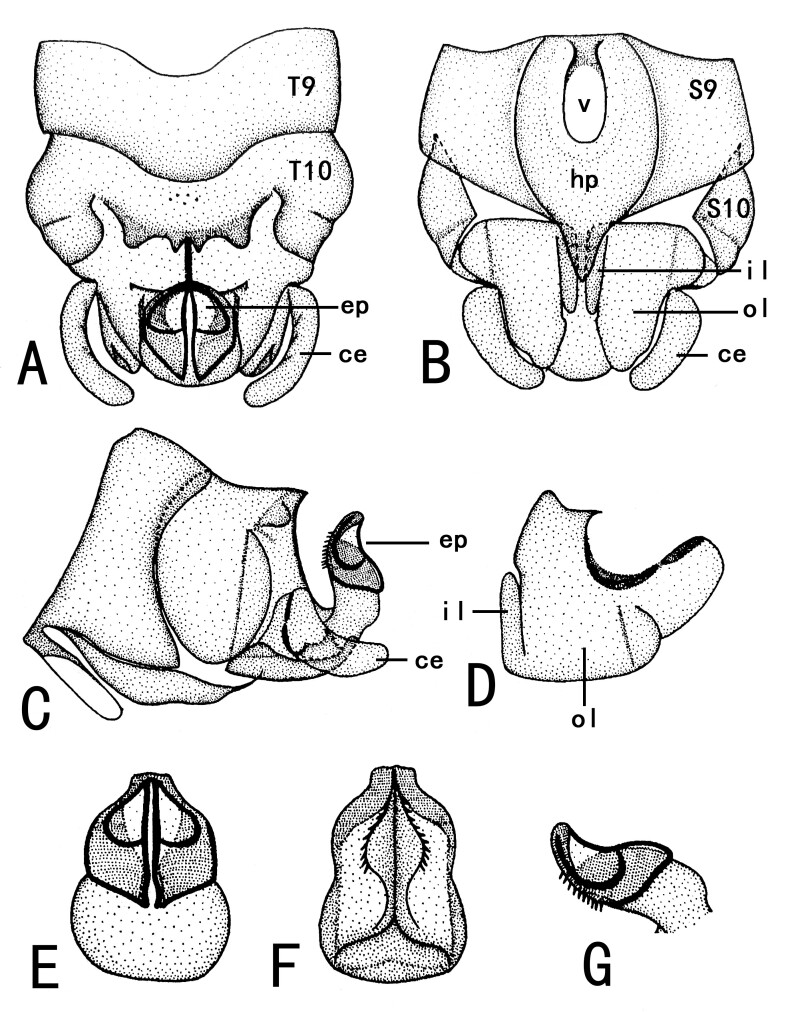
Male of *Illiesonemourabituberculata* sp. nov. **A** terminalia in dorsal view; **B** terminalia in ventral view; **C** terminalia in lateral view; **D** paraproct (left) in ventral view; **E** epiproct in dorsal view; **F** epiproct in ventral view; **G** epiproct in lateral view. Abbreviations: T9, T10: Tergum 9, 10; ce: cercus; S9, S10: Sternum 9, 10; hp: hypoproct; v: vesicle; il: inner lobe of paraproct; ol: outer lobe of paraproct.

**Male**. Head brown, antennae slightly brown (Fig. 2); pronotum paler than head, anterior margin wider than posterior margin. A single unbranched cervical gill as typical of the genus present on each side of mid-line (Fig. 1A). Wings subhyaline, fumose, veins brown. Forewing length ca. 6 mm, hind wing 5.5 mm. Legs brown (Fig. 2). Tergum 9 slightly sclerotised, extending posteriorly as a broad lobe over tergum 10. Tergum 10 slightly sclerotised anteriorly, but being more sclerotised along the posterior margin where a pair of lateral knobs and a pair of strong median prongs are present, both most noticeable in dorsal view, a field of 5-6 tiny spines present anteromedially (Fig. 1B). Hypoproct broad basally and tapering to a small, narrow tip, vesicle plump, length 2x width (Fig. 3B). Cerci mostly membranous, long thin and bent inwards (Fig. 1B, Fig. 3A-C). Paraprocts divided into two lobes; inner lobe slightly sclerotised, thin and long; outer lobe broad basally, large triangular, outer part curving dorsally, with outer margin darkly sclerotised and with outward directed sclerotised prong from outer margin near cerci (Fig. 3B and D). Epiproct short and broad in dorsal aspect; dorsal face with a pair of sclerotised bands each side of mid-line and distal margin of dorsal sclerite darkly sclerotised, extending anterolaterally, enveloping tip of ventral sclerite, which is more distinct in lateral and ventral views; basal sclerite membranous; ventral sclerite strongly sclerotised, broad basally and with lateral knobs at basolateral corners, becoming narrower towards apex, forming a pair of ridges each side of mid-line, each bearing a row of spines on anterior half (Fig. 3E-G).

#### Diagnosis

The male of this species is characterised by two pairs of small knobs arising posteromedially from tergum 10. They are similar to *I.tuberostyla* (Wu 1962) in the lateral view of the epiproct, both looking like tumours, but can be separated from the latter by the knobs protruding from the posterior margin of tergum 10. In *I.tuberostyla*, there was no mention of knobs on the terga.

#### Etymology

The name refers to the pair of knobs and strong prongs located posteromedially on tergum 10. The Latin adjective “*tuberculata*” means having knobs.

#### Distribution

China: Sichuan Province.

### Illiesonemoura motuoensis

Du & Ji sp. nov.

CF1BDE7A-F17C-5B83-A53B-D55F239316EF

E5E27817-3E84-46E1-83C5-53F8F91F9ADC

#### Materials

- **Type status:** Holotype. **Occurrence:** recordedBy: Du Yu-zhou; individualID: Insect collection of Yangzhou University (ICYZU), Jiangsu Province, China; individualCount: 1; sex: male; lifeStage: adult; occurrenceStatus: present; occurrenceID: 6B627470-E757-5D01-8D75-6DE223856414; **Taxon:** kingdom: Animalia; phylum: Arthropoda; class: Insecta; order: Plecoptera; family: Nemouridae; genus: Illiesonemoura; specificEpithet: *motuoensis*; **Location:** continent: Aisa; country: China; countryCode: CN; stateProvince: Tibet; county: Medog; locality: Hami-Beibeng; minimumElevationInMeters: 780; verbatimLatitude: 29.233333 N; verbatimLongitude: 95.166667 E; **Identification:** identifiedBy: Zhi-jie Wang, Yu-zhou Du & Xiao-yu Ji; **Event:** year: 2009; month: 6; day: 17; **Record Level:** language: en

#### Description

**Figure 4.**
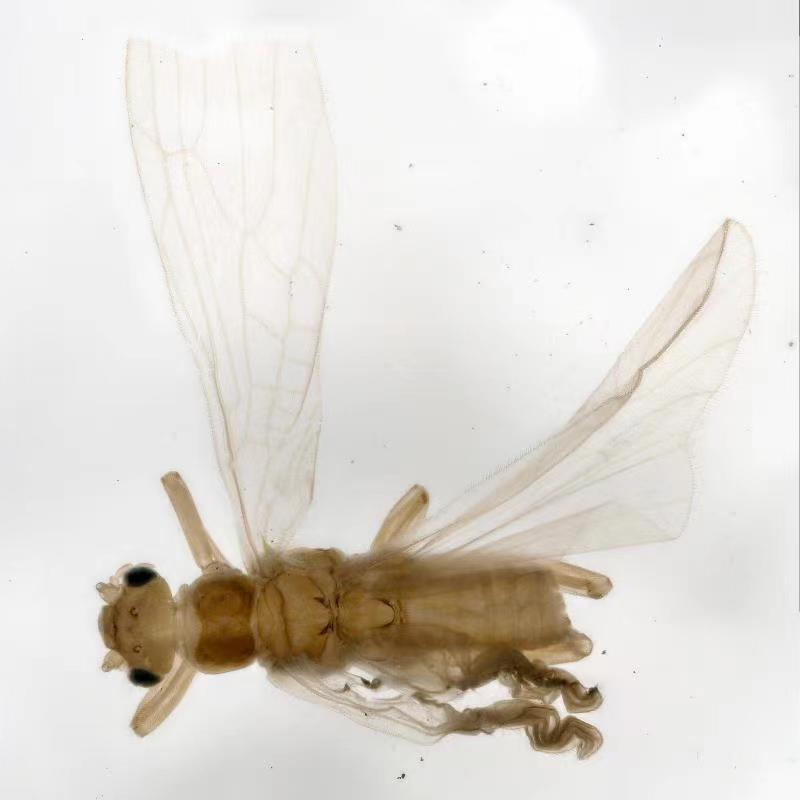
Male of *Illiesonemouramotuoensis* sp. nov. Adult, dorsal view.

**Figure 5.**
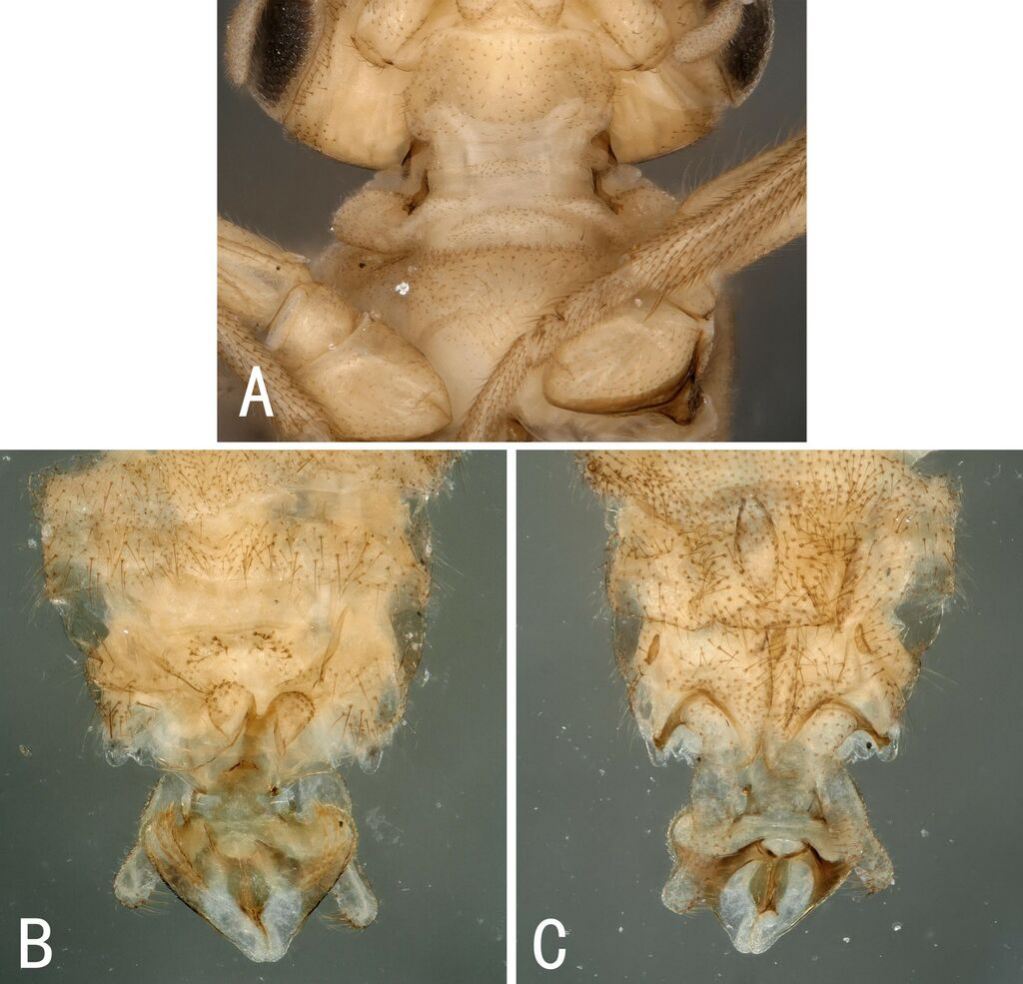
Male of *Illiesonemouramotuoensis* sp. nov. **A** cervical region in ventral view; **B** terminalia in dorsal view; **C** terminalia in ventral view.

**Figure 6.**
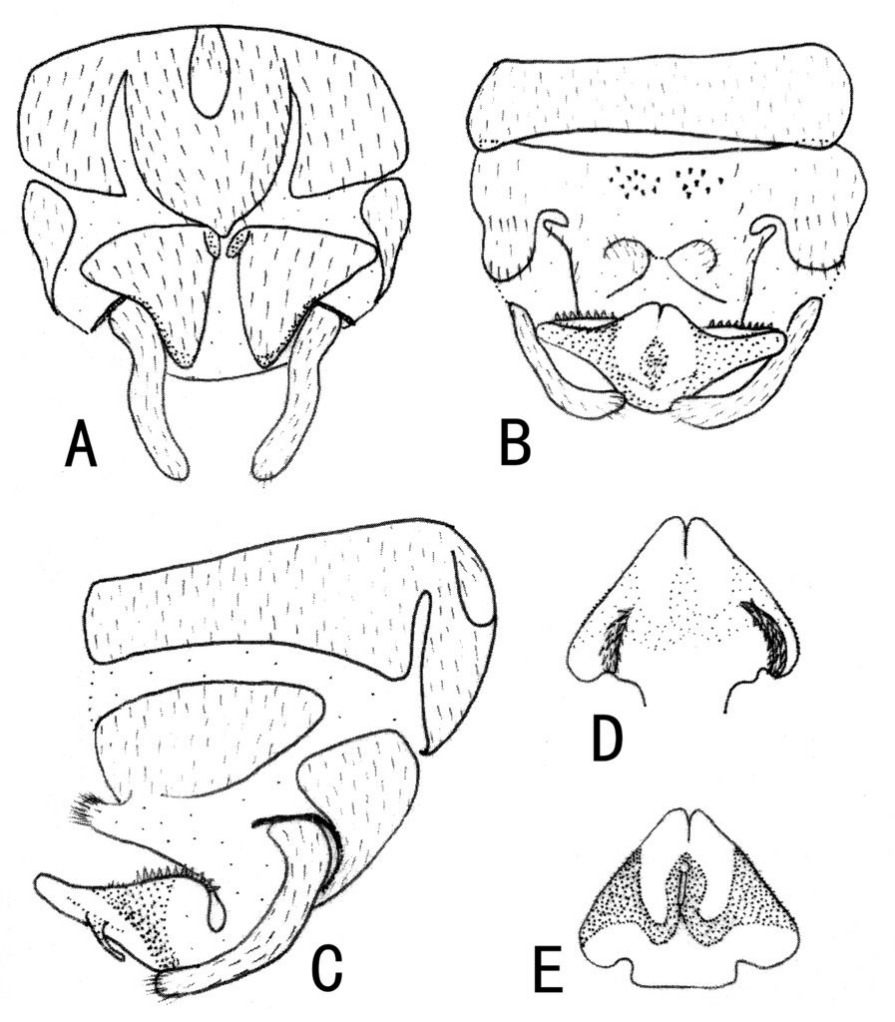
Male of *Illiesonemouramotuoensis* sp. nov. **A** terminalia in ventral view; **B** terminalia in dorsal view; **C** terminalia in lateral view; **D** epiproct in ventral view; **E** epiproct in dorsal view.

**Figure 7.**
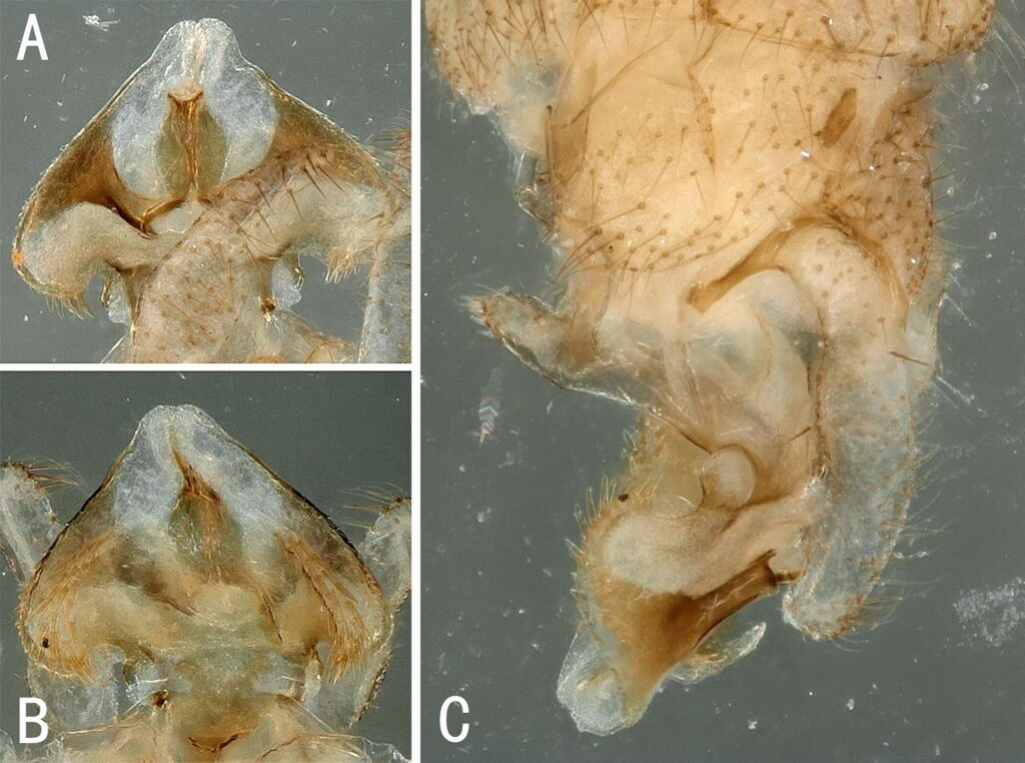
Male of *Illiesonemouramotuoensis* sp. nov. **A** epiproct in dorsal view; **B** epiproct in ventral view; **C** epiproct in lateral view.

**Male.** Head and antennae slightly brown; pronotum brown, angles blunt rounded (Fig. 4). A single unbranched cervical gill on each side of mid-line (Fig. 5A). Wings hyaline, veins brownish. Forewing length 4.8 mm, hind wing 4.0 mm. Legs brown. Abdomen pale with hairs. Tergum 10 bearing several small spines on anterior part and forming a pair of pale knobs medially, with hairs on the rounded tip (Fig. 5B and Fig. 6C). Hypoproct sub-quadrant with a small narrow tip; vesicle short, length 2x width. Cerci membranous, very long and thin with many hairs, upcurved in lateral view, sinuous in dorsal view (Fig. 6A-C). Paraprocts divided into 2 lobes; inner lobe slightly sclerotised, thin and short; outer lobe membranous mostly, broad basally, tapering to the apex, outer margin slightly sclerotised (Fig. 5C and Fig. 6A). Epiproct heart-shaped; dorsal sclerite notched apically, median part forming a pair of sclerotised bands, with a thin and slightly sclerotised protrusion between the sclerotised bands, outer margins of sclerotised bands bearing spinules; ventral sclerite with a pair of slightly sclerotised bands, each band bearing many spines (Fig. 6D-E and Fig. 7A-C).

#### Diagnosis

The male of this species is characterised by the heart-shaped epiproct with a thin and slightly sclerotised structure projecting between the sclerotised bands. In *I.tuberostyla* (Wu 1962) and *I.bituberculata* sp. nov., the epiprocts are tumour-shaped and there are no structures projecting from the epiproct.

#### Etymology

This species is named after the type locality, Medog County. “Motuo” is the spelling of “Medog” in Chinese pinyin.

#### Distribution

China: Tibet.

### Illiesonemoura weii

Du & Ji sp. nov.

6F2CC3A6-72CB-524C-ABDD-72599296529B

71B97B05-046F-4260-9CF8-6A154EB476D0

#### Materials

- **Type status:** Holotype. **Occurrence:** recordedBy: Yu-zhou Du; individualID: Insect collection of Yangzhou University (ICYZU), Jiangsu Province, China; individualCount: 1; sex: male; lifeStage: adult; occurrenceStatus: present; occurrenceID: B14BB907-D4A5-5748-876D-091C0125194E; **Taxon:** kingdom: Animalia; phylum: Arthropoda; class: Insecta; order: Plecoptera; family: Nemouridae; genus: Illiesonemoura; specificEpithet: *weii*; **Location:** continent: Aisa; country: China; countryCode: CN; stateProvince: Sichuan; locality: a suburb of Kangding City, river near Enwei Pharmaceutical Factory; minimumElevationInMeters: 3525; verbatimLatitude: 30.050000 N; verbatimLongitude: 101.566667 E; **Identification:** identifiedBy: Zhi-jie Wang, Yu-zhou Du & Xiao-yu Ji; **Event:** year: 2009; month: 7; day: 1; **Record Level:** language: en

#### Description

**Figure 8.**
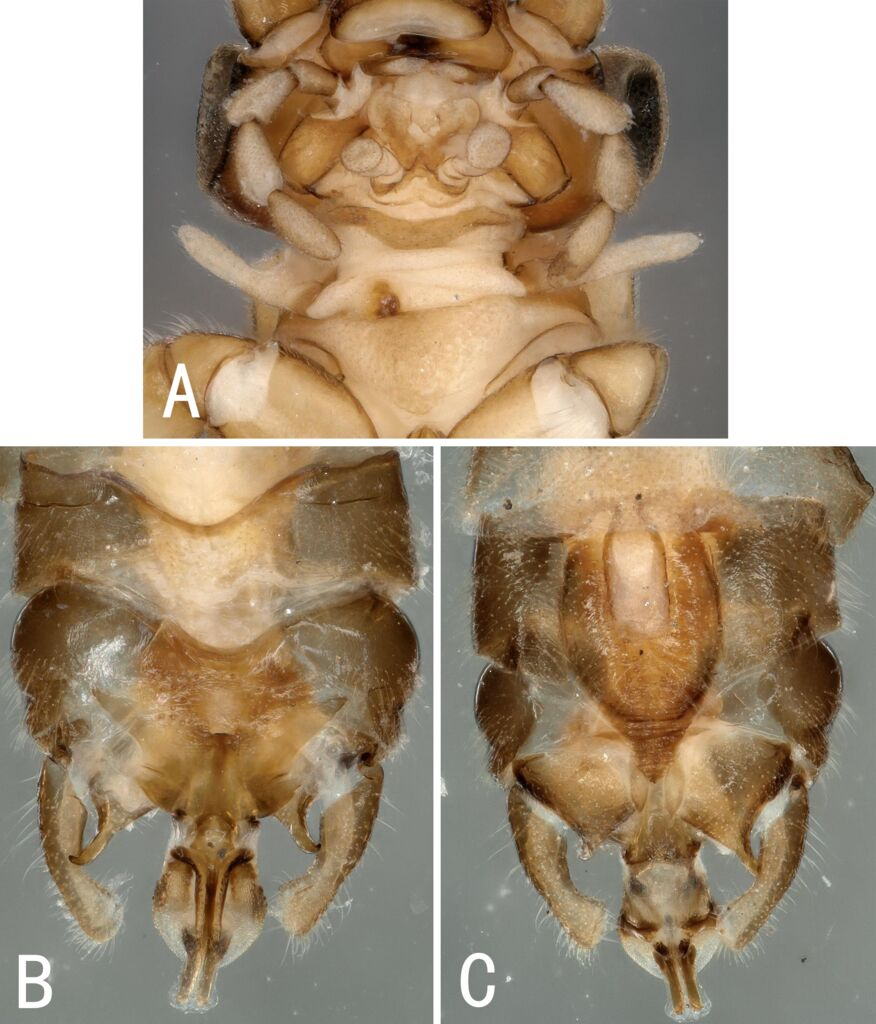
Male of *Illiesonemouraweii* sp. nov. **A** cervical region in ventral view; **B** terminalia in dorsal view; **C** terminalia in ventral view.

**Figure 9.**
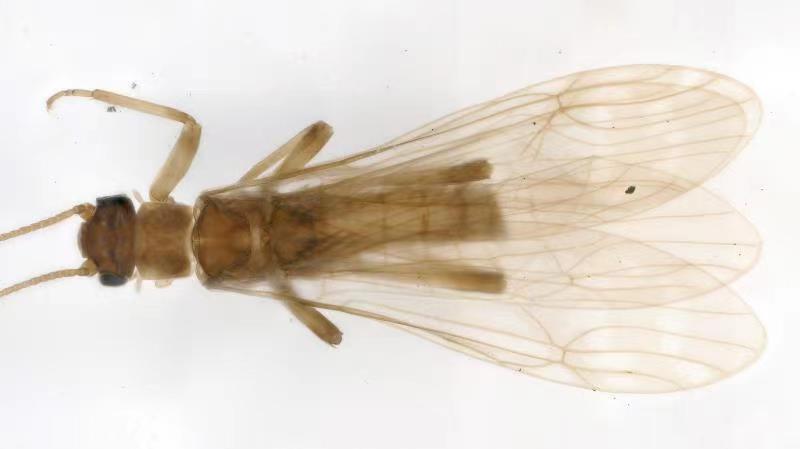
Male of *Illiesonemouraweii* sp. nov. Adult , dorsal view.

**Figure 10.**
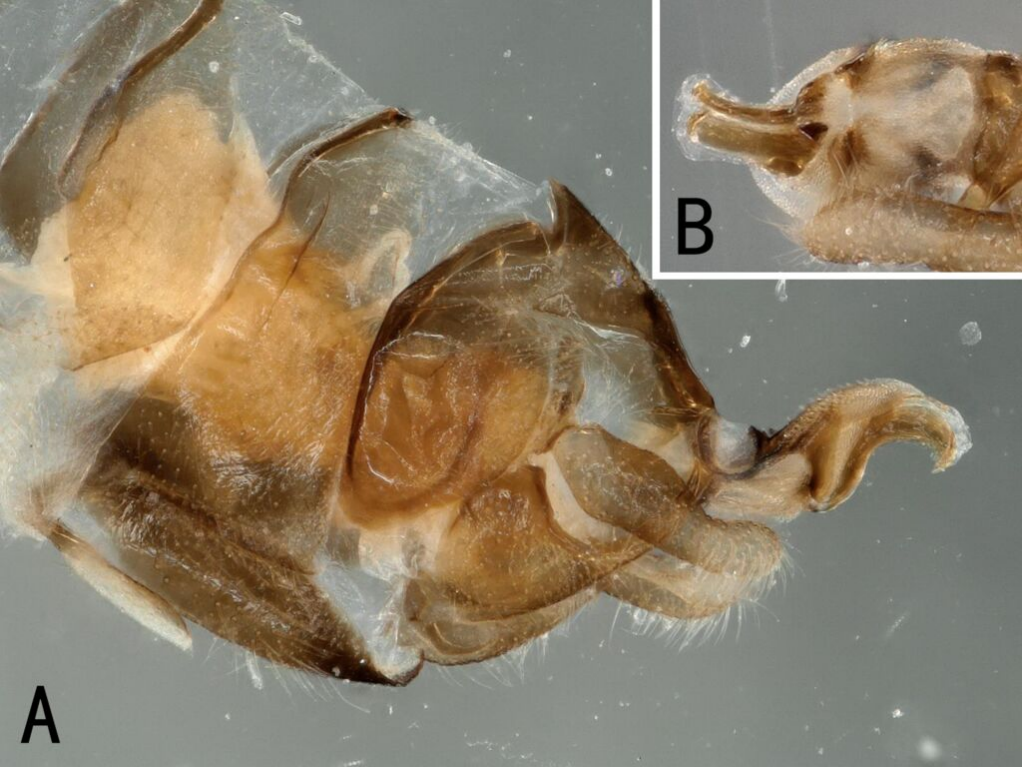
Male of *Illiesonemouraweii* sp. nov. **A** terminalia in lateral view; **B** epiproct in lateral view.

**Figure 11.**
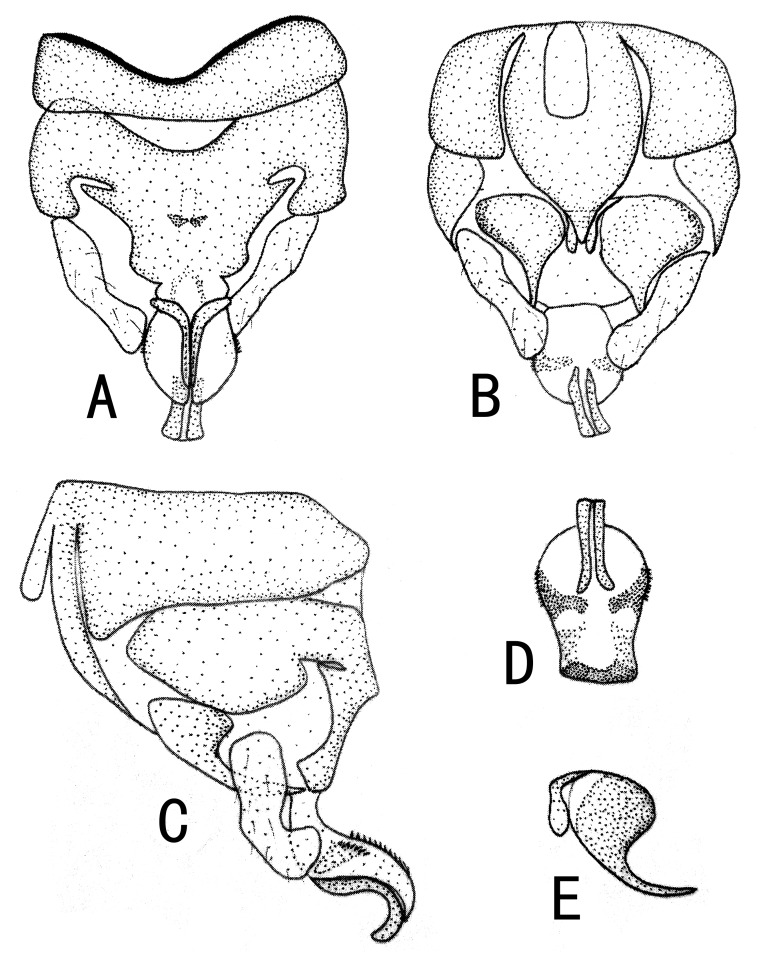
Male of *Illiesonemouraweii* sp. nov. **A** terminalia in dorsal view; **B** terminalia in ventral view; **C** terminalia in lateral view; **D** epiproct in dorsal view; **E** paraproct (left) in lateral view.

**Figure 12.**
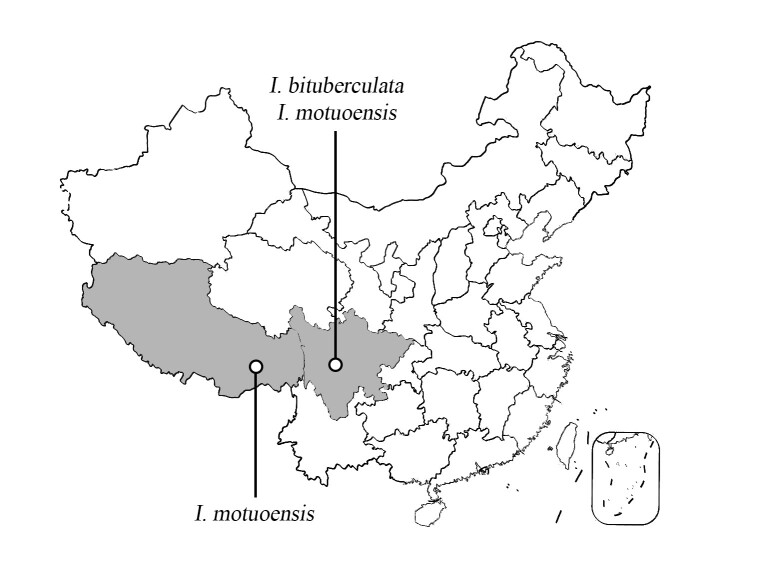
Distribution map of the three new species in China.

**Male.** Head brown, antennae slightly brown (Fig. 9); pronotum brown with dark brown stripes, anterior margin wider than posterior margin. A single unbranched cervical gill on each side of mid-line (Fig. 8A). Wings subhyaline, with light spots, veins brown. Forewing length 5.9 mm, hind wing length 5.0 mm. Legs brown, the middle of femora slightly brown. Anterolateral margin of terga 1–5 sclerotised. Terga 6-9 sclerotised anteriorly, but concave mid-anteriorly. Tergum 10 sclerotised, with a pair of small knobs near mid-length at each side of mid-line, which is more distinct in lateral view, with a small oval membranous portion above the knobs. Hypoproct broad basally and tapering to the tip; vesicle plump, rectangular, length 2x width. Cerci light brown, bent inwards and upwards, with hairs (Fig. 8B-C, Fig. 10A and Fig. 11A-C). Paraprocts divided into 2 lobes; inner lobe slightly sclerotised, short, sub-oval; outer lobe sclerotised, except inner margin, outer margin darkly sclerotised, broad basally, then slender, with a pointed tip (Fig. 11B and E). Epiproct short and broad, stretching back in side view, with the tip curved down; dorsal sclerite with a pair of sclerotised curved bands each side of mid-line, extending forward. Median part of epiproct with a pair of transverse sclerotised stripes, bearing spinules laterally; ventral sclerite slightly sclerotised, forming a pair of ridges each side of mid-line, each bearing a row of spines (Fig. 10B and Fig. 11D).

#### Diagnosis

The male of this species is characterised by a pair of small knobs from tergum 10, outer lobe of paraprocts with a pointed tip and epiproct bearing spinules laterally. It is similar to *I.tuberostyla* (Wu 1962) and *I.bituberculata* sp. nov. in the tumour-shaped epiproct. However, in *I.tuberostyla*, there is no portion extending from the epiproct anteriorly. It is similar to *I.bituberculata* in dorsal sclerite with a pair of sclerotised bent bands each side of mid-line. However, the length of the portion extending anteriorly is twice as long than that in *I.bituberculata*. Median part of dorsal sclerite in *I.weii* bears a pair of transverse sclerotised stripes, forming spinules laterally, which are absent in *I.bituberculata*.

#### Etymology

This new species is named in honour of Professor Wei Mei-Cai (Central South University of Forestry and Technology, Changsha, Hunan, China), a taxonomist of Chinese sawfly (Hymenoptera, Symphyta) for organising the biodiversity expeditions in Tibet, Qinghai and Sichuan.

#### Distribution

China: Sichuan Province.

## Supplementary Material

XML Treatment for Illiesonemoura bituberculata

XML Treatment for Illiesonemoura motuoensis

XML Treatment for Illiesonemoura weii

## References

[B10411059] Aubert J (1959). P1écoptères du Pakistan. Mémoires de la Société Vaudoise des Sciences Naturelles.

[B10411068] Baumann R W (1975). Revision of the stonefly family Nemouridae (Plecoptera): A study of the world fauna at the generic level.. Smithsonian Contributions to Zoology.

[B10411078] Harper P P (1975). *Amphinemura* et *Nemoura* nouvelles du Nepal (Plécoptères: Némouridés). Nouvelle Revue d’ Entomologie.

[B10411087] Jewett S G (1958). Entomological survey of the Himalaya, Part 23: Stoneflies (Plecoptera) from the north-west (Punjab) Himalaya. Proceedings of the National Academy Science (India).

[B10411096] Kawai T (1968). Stoneflies (Plecoptera) from Taiwan in the Bishop Museum, Honolulu. Pacific Insects.

[B10411105] McLachlan R (1875). Neuroptera. In: Fedchenko AP (Ed) Travel to Turkestan. Zoogeographical studies,.

[B10411114] Mo R R, Gamboa M, Watanabe K, Wang G Q, Li W H, Yang D, Murányi D (2020). A remarkable new genus and species of Nemourinae (Plecoptera, Nemouridae) from Sichuan, China, with systematic notes on the related genera. PLoS One.

[B10632542] RE DeWalt, H Hopkins, U Neu-Becker, G Stueber Plecoptera Species File.. http://plecoptera.speciesfile.org/.

[B10411135] Wu C F (1962). Results of the Zoologico-Botanical Expedition to Southwest China, 1955-1957 (Plecoptera). Acta Entomologica Sinica.

[B10411039] Zhao M Y, Abdur R, Du Y Z (2023). New species of *Illiesonemoura* and *Nemoura* (Plecoptera: Nemouridae) from Yunnan Province of southern China.. Zootaxa.

